# In-depth analysis of risk factors for postoperative pulmonary infection in patients with basal ganglia haemorrhage and construction of prediction model: based on domestic and international cutting-edge clinical research and big data analysis

**DOI:** 10.3389/fmed.2025.1627298

**Published:** 2025-07-23

**Authors:** Min Chen, Longbiao Da, Chun Huang, Jie Liu, Jian Tang, Zhengjiang Zha

**Affiliations:** Department of Neurosurgery, East Campus of Anqing Municipal Hospital, Anqing, China

**Keywords:** basal ganglia haemorrhage, lung infection, smoking, GCS score, predictive model

## Abstract

**Background:**

Basal ganglia haemorrhage is a common and serious cerebrovascular disease with a high rate of disability and mortality. Postoperative patients often face many complications, among which pulmonary infection is particularly prominent. Lung infections not only significantly prolong patients’ hospital stay and increase healthcare costs, but also greatly affect the prognostic regression of patients, and may even lead to a rapid deterioration of the condition, which is one of the most important causes of death in patients with basal ganglia haemorrhage.

**Objective:**

To investigate the high-risk factors for the development of postoperative pulmonary infections in patients with basal ganglia haemorrhage and to develop a predictive model.

**Methods:**

A total of 317 patients were collected in this study, of which 126 patients developed postoperative lung infections; the patients enrolled in this study were randomly divided into a training set and a validation set according to the ratio of 7:3, of which 221 were in the training set and 96 were in the validation set. Past medical history, smoking and alcohol consumption, and relevant information during hospitalisation were collected separately to study the correlation factors affecting the emergence of postoperative lung infection in patients, and to establish a prediction model.

**Results:**

The potentially relevant factors were included in a one-way logistic regression and after analysing the results, a history of smoking, duration of ventilator use, preoperative tracheal intubation, preoperative vomiting, and preoperative GCS (Glasgow Coma Scale) scores were identified as potential risk factors for the development of postoperative pulmonary infections in patients with basal ganglia haemorrhage, *p* < 0.2; The data obtained were further included in a multifactorial review, and smoking history, duration of ventilator use, preoperative tracheal intubation, preoperative vomiting, and preoperative GCS scores were independent risk factors for the development of postoperative pulmonary infections in patients with basal ganglia haemorrhage, *p* < 0.05.

**Conclusion:**

The prediction model derived from this study provides a powerful tool for clinicians to identify patients at high risk of postoperative lung infection at an early stage.

## 1 Introduction

Basal ganglia haemorrhage accounts for a high proportion of cerebrovascular diseases, and as a common and serious acute condition, its pathogenesis is complex (1–3). The vascular structure of this region is special, and the bean artery branches out from the middle cerebral artery at a right angle, the diameter of the tube suddenly becomes thin and bears a larger pressure, and the wall of the blood vessel is prone to be damaged and ruptured under the pathological state of hypertension and atherosclerosis, which triggers haemorrhage. A large number of clinical studies have shown that basal ganglia haemorrhage is extremely rapid in onset, with a high degree of risk, and the disability and fatality rates of patients are always at a high level, which brings a heavy burden to the patients’ families and the society (4–6). Postoperatively, patients with basal ganglia haemorrhage are severely challenged by a variety of complications, of which pulmonary infection is one of the most problematic and highly prevalent (7, 8). Lung infections, when present, can lead to a range of serious consequences (9). In the short term, patients’ hospitalisation time is greatly extended, their bodies have to endure the discomfort caused by the infection, and the psychological stress may increase, while the medical cost rises significantly, resulting in a large consumption of medical resources. From a long-term perspective, lung infection seriously hinders the recovery of nerve function, interferes with the repair and remodelling of nerve conduction pathways, and affects the rehabilitation process of limb movement, speech and other functions, which has a great negative impact on the overall prognosis. In some patients with serious conditions, lung infection may even be the direct cause of death (7, 10–12).

Scholars at home and abroad have conducted a large number of studies around postoperative lung infection after basal ganglia haemorrhage, and have made some progress in risk factor analysis and prediction model construction (13, 14). For example, Ruihong et al. found that age, nasogastric tube, and neutrophil-to-lymphocyte ratio had high predictive value for stroke-related pneumonia. However, their study included fewer risk factors, and risk factors related to the patient’s general condition, such as hypertension, diabetes, and surgical status, were not included in the study (15). Folin et al. constructed a predictive model for secondary venous thromboembolism in stroke patients based on factors such as pulmonary infection and partial thromboplastin time. Although their study covered multiple systems, including the circulatory and respiratory systems, they did not conduct research on the correlation between stroke and pulmonary infection (16). Although Yang et al. modelled postoperative pulmonary infection after stroke, the variability of the model was complex and could not be fully quantified and analysed (17). In addition, Factors such as age, underlying diseases (e.g., diabetes, Chronic Obstructive Pulmonary Disease, etc.), length of surgery, and duration of mechanical ventilation are known to be associated with lung infections, and a number of predictive models have been proposed (14). However, basal ganglia haemorrhage involves complex pathophysiological changes in multiple systems such as neurological, vascular and immune systems, and the interaction between various factors makes the existing studies still have ambiguities in the precise determination of risk factors, and the accuracy and universality of the prediction models also need to be improved. For example, some models do not fully consider the differences in the underlying disease spectrum of patients in different regions, which limits their effectiveness in clinical application (18). In view of this, this study will comprehensively and deeply analyse the risk factors of postoperative lung infection in patients with basal ganglia haemorrhage, integrate multicentre data, and use advanced statistical methods and machine learning algorithms to construct a more accurate and efficient prediction model with wide applicability, so as to provide a solid basis for the early and precise identification of high-risk patients in the clinic, and the formulation of personalised intervention strategies.

## 2 Materials and methods

### 2.1 Research objectives

This study was conducted on 317 patients with basal ganglia haemorrhage, 194 males and 123 females, who were admitted to our department for surgical treatment from January 2022 to December 2023. This study fulfils the relevant criteria of the Declaration of Helsinki.

### 2.2 Research methods

In this retrospective case–control study, a total of 317 patients were collected, of which 126 patients presented with postoperative lung infections. Information on past medical history, smoking and alcohol consumption, and hospitalisation was collected to study the correlation factors affecting the development of postoperative lung infections in patients with basal ganglia haemorrhage.

### 2.3 Collecting indicators

Gender, age, history of smoking, history of alcohol consumption, hypertension, coronary artery disease, diabetes mellitus, preoperative GCS (Glasgow Coma Scale) score, haematoma volume, duration of ventilator use, preoperative endotracheal intubation, and incidence of preoperative vomiting were collected from the patients in this study. These data were collected by trained clinicians in the Department of Neurosurgery at our hospital between January 2022 and December 2023. All data were obtained from medical records.

### 2.4 Handling of missing data

Some variables in the study subjects may have missing data. If a study subject has missing data in two or fewer variables, calculate the mean value of each variable to impute the missing data. If a study subject has missing data in more than two variables, it is considered to have incomplete clinical data, and the study subject is excluded according to the exclusion criteria.

### 2.5 Inclusion and exclusion criteria

#### 2.5.1 Inclusion criteria

① It meets the diagnostic criteria of China stroke surveillance report 2021 (19). ② The site of haemorrhage is located in the basal ganglia region as diagnosed by imaging. ③ Age ≥ 18 years old. ④Patients (family members) gave informed consent and participated voluntarily. ⑤ Complete clinical information.

#### 2.5.2 Exclusion criteria

① Combined with other organ trauma, severe shock, etc. ② Combined with other diseases, such as malignant tumours, blood diseases; ③ those with severe mental illness or patients who died during the study; ④ Those who are receiving other treatments or participating in other clinical studies. ⑤ The research subjects had more than two missing variables.

### 2.6 Interpretation of some validation indicators mentioned in this study

(1) Necessity of ventilator in patients with basal ganglia haemorrhage: patients with basal ganglia haemorrhage are often accompanied by consciousness disorders and coma, and when the volume of haemorrhage is large, midline displacement as well as compression of respiratory centre can occur, so when the volume of basal ganglia haemorrhage, consciousness disorders are serious, or secondary respiratory failure, the ventilator is a key means of life-saving.
(2) The need for preoperative tracheal intubation in patients with basal ganglia haemorrhage: The core indications for preoperative tracheal intubation in patients with haemorrhage in the basal ganglia region are that the patient is severely impaired in consciousness, has respiratory failure or that the patient requires emergency surgery. For awake or mildly unconscious patients undergoing elective surgery, a non-intubated option is available under close monitoring, balancing the risks of anaesthesia with the protection of brain tissue.
(3) Preoperative GCS: In this study, GCS scores were assessed in the study subjects prior to anaesthetic induction. The GCS score includes three aspects: Eye Opening, verbal response, and motor response.
  Eye Opening: From 1 to 4 points: no eye opening response, eye opening in response to pain stimulation, eye opening in response to verbal stimulation, spontaneous eye opening.
  Verbal response: From 1 to 5 points: No verbal response, only able to vocalise, inappropriate speech, confused speech but able to communicate, normal orientation.
  Motor response: From 1 to 6 points: no movement response, abnormal flexion, pain on extension, pain on flexion, pain localisation, specified movements.
  Preoperative GCS score calculation: eye opening + verbal response + motor respo. Range: 3–15 points.
(4) Postoperative pulmonary infection: Postoperative pulmonary infection was defined in accordance with the surveillance definition provided by the Centres for Disease Control and Prevention and the National Healthcare Safety Network (20).

### 2.7 Statistical methods

Data were processed and statistically analysed in this study using SPSS 25.0. This study uses R language for graphing. Quantitative information that conformed to normal distribution was expressed as mean ± standard deviation, and differences between groups were analysed using the independent samples t-test. Comparisons between groups that did not obey normal distribution were analysed using non-parametric tests. Data for qualitative information were expressed as number of cases and percentages, and the chi-square test was used to determine whether differences existed between groups. The patients were first divided into training and validation sets in a 7:3 ratio at random, and then analysed on the basis of the postoperative development of pulmonary infections in the patients in the training set and various clinically relevant indexes and other factors, and then based on the one-way logistic regression analysis of the collected data, the potential risk factors for the development of pulmonary infections in patients with basal ganglia haemorrhage in the postoperative period were determined. For the univariate analysis, exposure factors with *p* ≤ 0.2 were selected and included in the multivariate analysis (21, 22). An independent risk factor for developing postoperative pulmonary infection in patients with basal ganglia haemorrhage was derived, with *p* < 0.05 being considered a statistically significant difference. Subsequent internal validation of the model further confirmed the reliability of the predictive model derived in this study.

## 3 Results

### 3.1 Table of values of relevant indicators for this study

This study assigns values to the variables. Group without lung infection, female, non-smoking, non-drinking, no history of hypertension, no history of coronary heart disease, no history of diabetes mellitus, haematoma volume less than 30 mL, ventilator use less than 24 h, no tracheal intubation and no history of preoperative vomiting are assigned to 0. The other cases of the variable are assigned a value of 1 (Table 1).

**Table 1:** Assignment of values to relevant indicators.

Name	Variable assignment and description
Group	Group without lung infection-0, Lung Infection Group-1
Gender	Female-0, Male-1
History of smoking, alcohol consumption	No-0, Yes-1
History of hypertension, Diabetes, coronary heart disease	No-0, Yes-1
Haematoma volume	<30 ml-0, ≥ 30 mL-1
Ventilator use time	<24 h-0, ≥ 24 h-1
Preoperative tracheal intubation	No-0, Yes-1
preoperative vomiting	No-0, Yes-1

### 3.2 Comparison of baseline features between training and validation sets

We randomly divided the patients included in this experimental study into a training set and a validation set in a ratio of 7:3. Among them, 221 were in the training set and 96 were in the validation set. The general information of the patients in the two sets was included in the statistical study, *p* > 0.05, and the differences in the baseline characteristics of the two sets were not statistically significant, as shown in Table 2.

**Table 2:** Comparison of baseline features between training and validation sets.

Variables	Total (*n* = 317)	Test (*n* = 96)	Train (*n* = 221)	Statistic	*p*
Age	59.52 ± 11.96	59.18 ± 12.35	59.67 ± 11.82	t = −0.34	0.734
Preoperative GCS score	6.55 ± 2.38	6.44 ± 2.41	6.60 ± 2.38	t = −0.55	0.584
Group, n(%)				χ^2^ = 2.92	0.087
0	191 (60.25)	51 (53.12)	140 (63.35)		
1	126 (39.75)	45 (46.88)	81 (36.65)		
Gender, n(%)				χ^2^ = 0.32	0.573
0	123 (38.80)	35 (36.46)	88 (39.82)		
1	194 (61.20)	61 (63.54)	133 (60.18)		
History of smoking n(%)				χ^2^ = 1.14	0.286
0	163 (51.42)	45 (46.88)	118 (53.39)		
1	154 (48.58)	51 (53.12)	103 (46.61)		
History of alcohol consumption, n(%)				χ^2^ = 0.72	0.395
0	207 (65.30)	66 (68.75)	141 (63.80)		
1	110 (34.70)	30 (31.25)	80 (36.20)		
Hypertension, n(%)				χ^2^ = 0.08	0.777
0	49 (15.46)	14 (14.58)	35 (15.84)		
1	268 (84.54)	82 (85.42)	186 (84.16)		
Diabetes mellitus, n(%)				χ^2^ = 0.71	0.401
0	269 (84.86)	79 (82.29)	190 (85.97)		
1	48 (15.14)	17 (17.71)	31 (14.03)		
Coronary Heart Disease, n(%)				χ^2^ = 0.95	0.329
0	297 (93.69)	88 (91.67)	209 (94.57)		
1	20 (6.31)	8 (8.33)	12 (5.43)		
Volume of haematoma, n(%)				χ^2^ = 0.02	0.896
0	45 (14.20)	14 (14.58)	31 (14.03)		
1	272 (85.80)	82 (85.42)	190 (85.97)		
Ventilator use time, n(%)				χ^2^ = 0.01	0.922
0	140 (44.16)	42 (43.75)	98 (44.34)		
1	177 (55.84)	54 (56.25)	123 (55.66)		
Preoperative tracheal intubation, n(%)				χ^2^ = 0.88	0.348
0	189 (59.62)	61 (63.54)	128 (57.92)		
1	128 (40.38)	35 (36.46)	93 (42.08)		
Preoperative vomiting, n(%)				χ^2^ = 0.32	0.572
0	141 (44.48)	45 (46.88)	96 (43.44)		
1	176 (55.52)	51 (53.12)	125 (56.56)		

### 3.3 Single factor analysis

The training set of 221 patients was included in the statistical analysis, of which 81 patients had postoperative lung infections. Possibly relevant factors were included in one-way logistic regression, in which smoking history, duration of ventilator use, preoperative endotracheal intubation, preoperative vomiting, and preoperative GCS scores were potential risk factors for the development of postoperative lung infections in patients with basal ganglia haemorrhage, *p* < 0.2. See Table 3 for details.

**Table 3:** One-factor logistic regression analysis based on training set.

Variables	*β*	S. E	Z	*p*	OR (95%CI)
Gender					
0					1.00 (Reference)
1	0.02	0.29	0.07	0.942	1.02 (0.58–1.79)
History of smoking					
0					1.00 (Reference)
1	0.57	0.28	2.02	0.043	1.77 (1.02–3.07)
History of alcohol consumption					
0					1.00 (Reference)
1	−0.03	0.29	−0.09	0.926	0.97 (0.55–1.72)
Hypertension					
0					1.00 (Reference)
1	−0.17	0.38	−0.45	0.654	0.84 (0.40–1.77)
Diabetes mellitus					
0					1.00 (Reference)
1	−0.23	0.41	−0.55	0.585	0.80 (0.36–1.79)
Coronary Heart Disease					
0					1.00 (Reference)
1	−0.58	0.68	−0.85	0.395	0.56 (0.15–2.13)
Volume of haematoma					
0					1.00 (Reference)
1	0.06	0.40	0.15	0.884	1.06 (0.48–2.34)
Ventilator use time					
0					1.00 (Reference)
1	0.89	0.29	3.03	0.002	2.44 (1.37–4.34)
Preoperative tracheal intubation					
0					1.00 (Reference)
1	0.87	0.29	3.06	0.002	2.40 (1.37–4.20)
Preoperative vomiting					
0					1.00 (Reference)
1	1.01	0.30	3.38	<0.001	2.75 (1.53–4.94)
Age	0.00	0.01	0.24	0.809	1.00 (0.98–1.03)
Preoperative GCS score	−0.38	0.07	−5.13	<0.001	0.69 (0.60–0.79)

### 3.4 Multi-factor analysis

The five risk factors derived from the univariate analysis of this study were further included in the multivariate analysis, and the results showed that history of smoking, duration of ventilator use, preoperative endotracheal intubation, preoperative vomiting, and preoperative GCS scores were the independent risk factors for the development of postoperative pulmonary infections in patients with basal ganglia haemorrhage. The details are shown in Table 4.

**Table 4:** Multi-factor Logistic regression analysis based on training set.

Variables	*β*	S. E	Z	*p*	OR (95%CI)
History of smoking					
0					1.00 (Reference)
1	1.17	0.35	3.33	<0.001	3.22 (1.62–6.43)
Ventilator use time					
0					1.00 (Reference)
1	1.05	0.36	2.94	0.003	2.86 (1.42–5.76)
Preoperative tracheal intubation					
0					1.00 (Reference)
1	0.71	0.35	2.02	0.044	2.03 (1.02–4.04)
Preoperative vomiting					
0					1.00 (Reference)
1	1.26	0.37	3.38	<0.001	3.52 (1.70–7.29)
Preoperative GCS scores	−0.38	0.08	−4.82	<0.001	0.68 (0.58–0.80)

### 3.5 Plotting of nomograms

**Figure 1:**
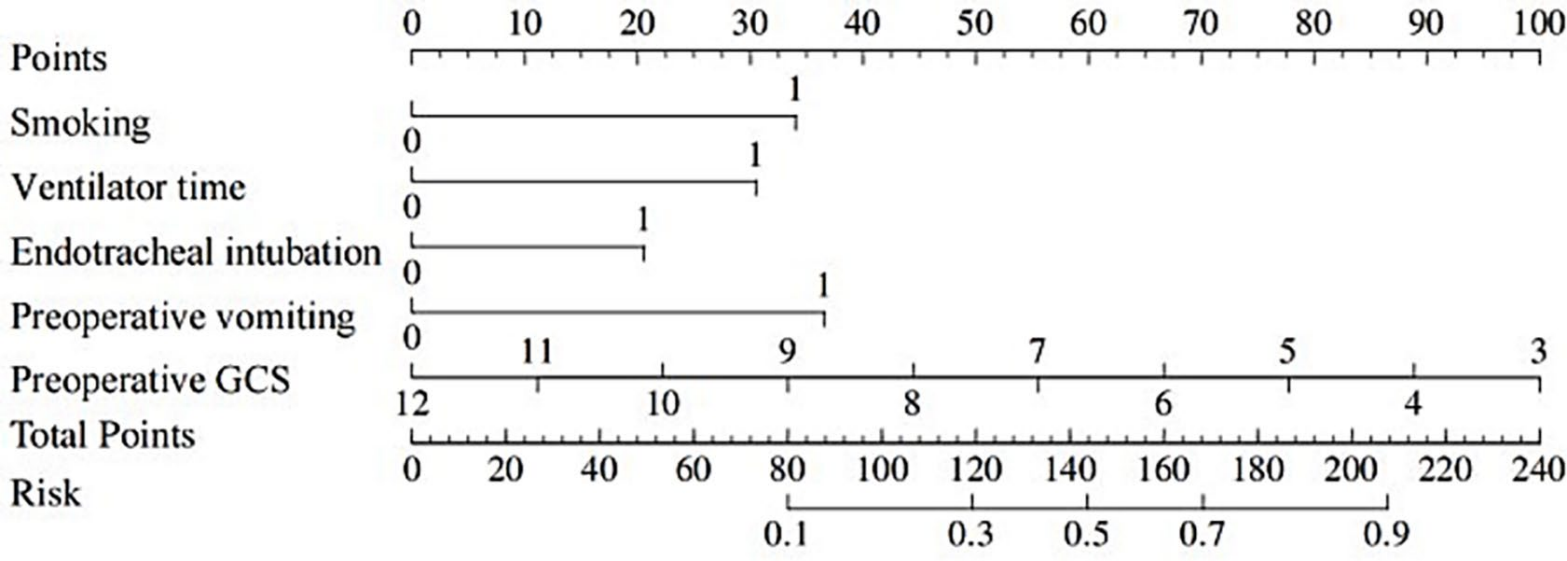
Nomogram prediction of the risk of developing postoperative pulmonary infection in patients with basal ganglia haemorrhage.

**Figure 2:**
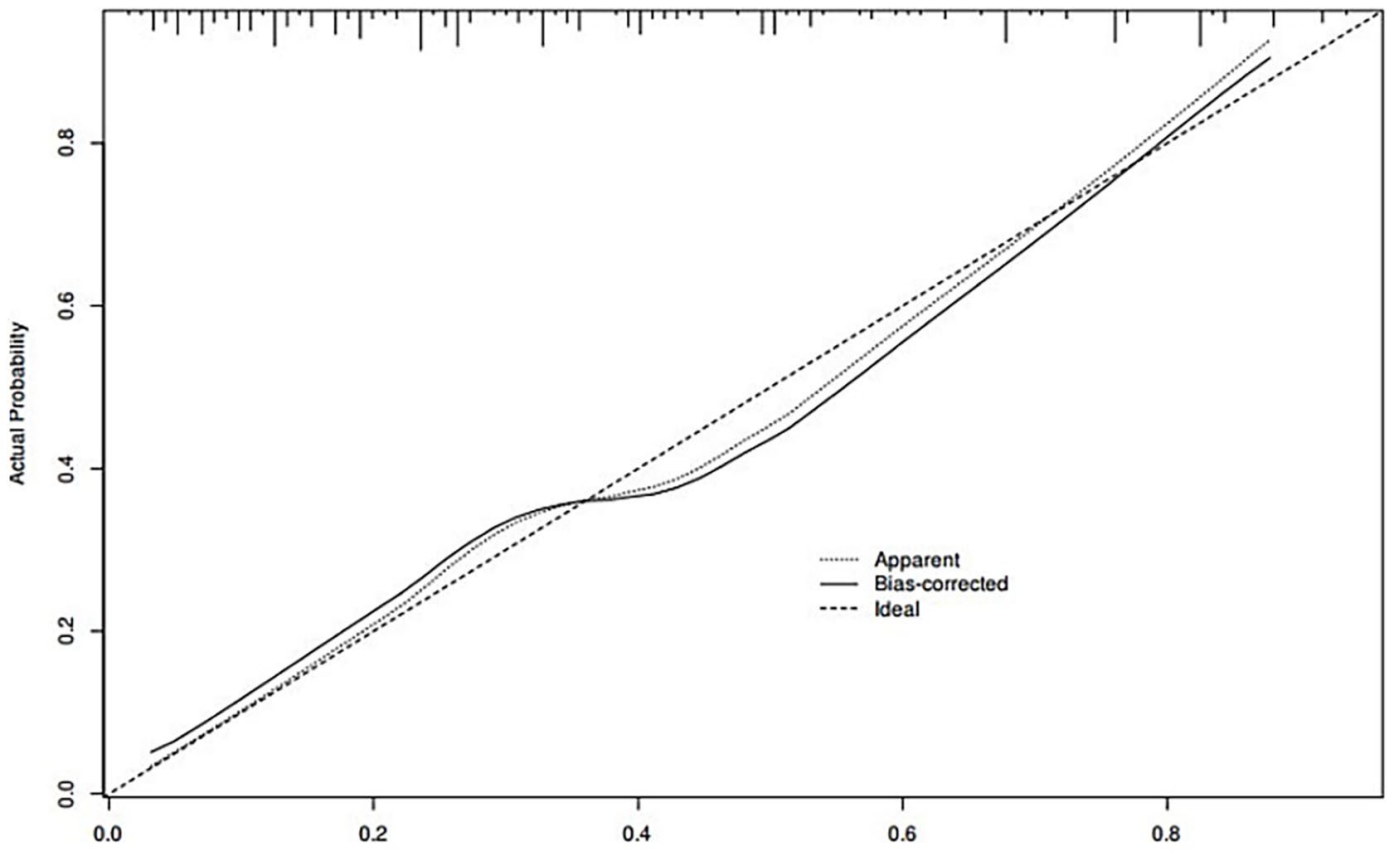
Internal validation of nomograms: calibration curves.

**Figure 3:**
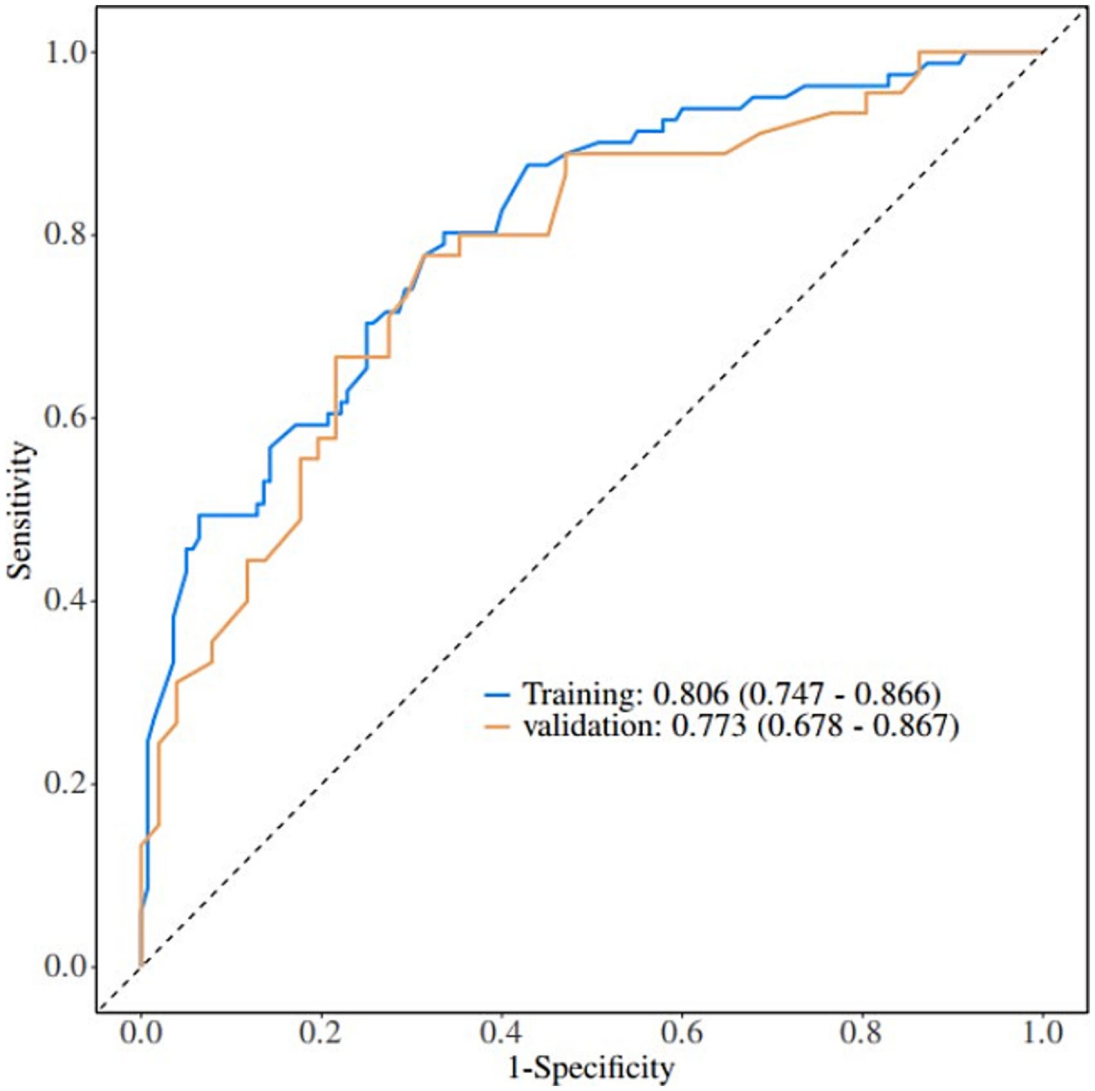
Validation of nomograms: ROC curves. ROC, Receiver operating characteristic.

**Figure 4:**
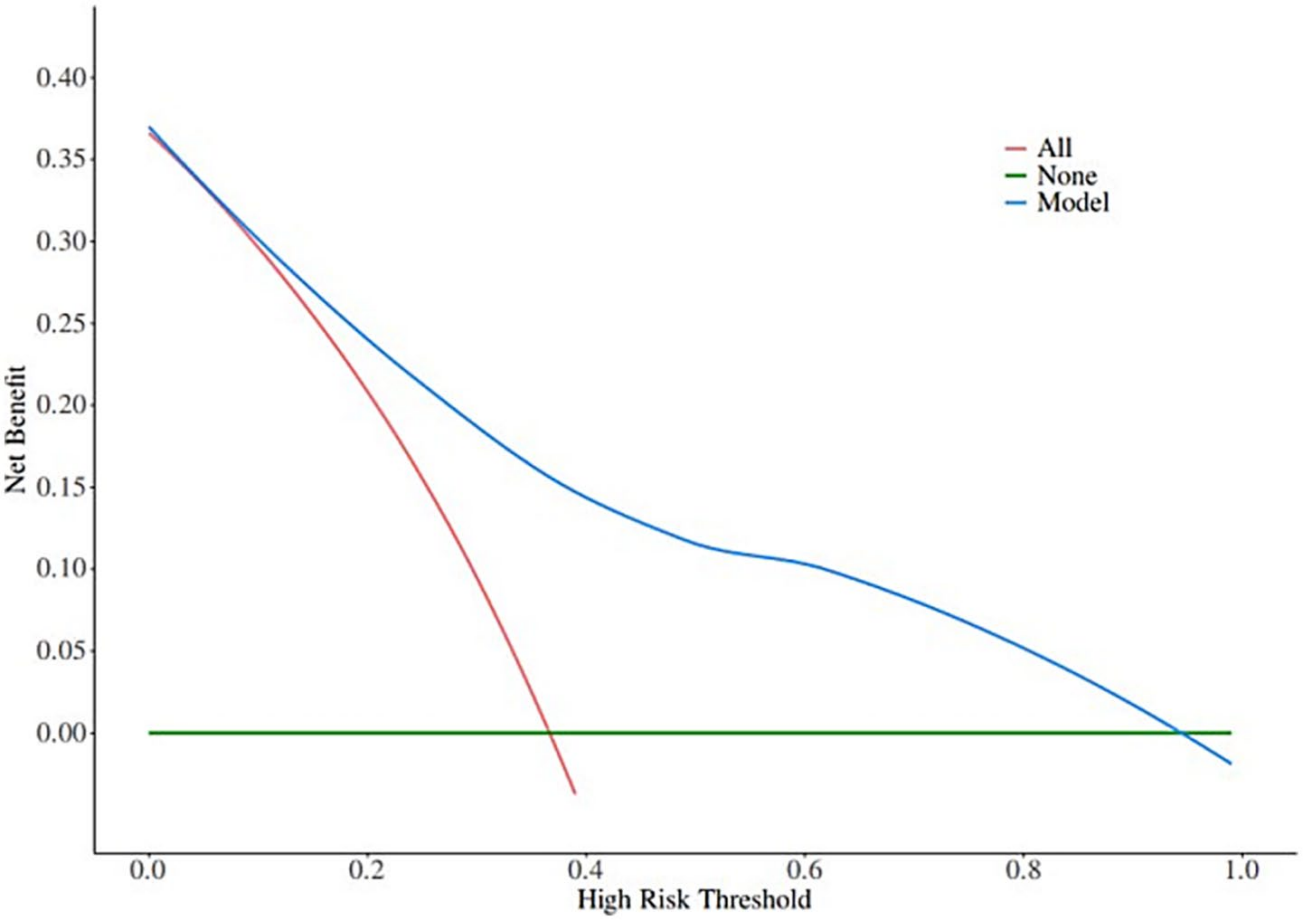
Decision curves in nomogram models.

A nomogram of the risk of developing postoperative pulmonary infections in patients with basal ganglia haemorrhage was constructed based on five independent predictors tested by multifactorial logistic regression analysis, as shown in Figure 1. Nomo score was assigned to each independent risk factor. Summing based on the clinical characteristics of this patient yields a total score, positioned on the Total points axis. The value on the Risk axis corresponding vertically downwards is the probability of developing a postoperative lung infection in that patient with a basal ganglia haemorrhage. The score for each independent predictor corresponds to the upper limit of the score for each independent predictor. The total score for each subject was the sum of each independent predictor score. The probability of developing a postoperative lung infection was determined by the total score on the axis of risk of developing a postoperative lung infection in patients with basal ganglia haemorrhage. The model was subsequently validated internally, and the internal validation was carried out by repeating the sampling of the nomograms 1,000 times using the Bootstrap method in the R software. The calibration curve is close to the ideal curve, indicating that the nomogram predicts the incidence of postoperative pulmonary infections in patients with basal ganglia haemorrhage with a high degree of agreement with the actual incidence, reflecting a good predictive performance, see Figure 2. The ROC curve for this nomogram training set, with an AUC of 0.888 (95% CI = 0.846–0.929); and the validation set ROC curve, with an AUC of 0.853 (95% CI = 0.780–0.925), is shown in Figure 3. This nomogram is shown to be a good discriminator between patients with basal ganglia haemorrhage who are at high risk of developing postoperative lung infections. The decision curve (DCA) for this nomogram shows that the model provides more net benefits than the ‘all intervene’ or ‘none intervene’ strategies when the threshold probability of an individual is greater than 0.05 in this column. This finding suggests that the nomogram model has good clinical utility in predicting the development of postoperative lung infections in patients with basal ganglia haemorrhage, see Figure 4.

## 4 Discussion

The management of postoperative pulmonary infections in patients with basal ganglia haemorrhage has always been a difficult challenge in clinical practice. Although previous studies have revealed the association between conventional factors such as age and underlying diseases, these results are still insufficient in the face of complex and changing clinical scenarios. After surgery for basal ganglia haemorrhage, patients often suffer from multiple complications, which seriously affect the recovery process and prognosis. Pulmonary infections, as one of the most difficult and frequent complications, have been the focus of clinical attention due to their high incidence and lethality. Existing studies have explored some of these complications, but there are still many gaps in the analysis and prediction of risk factors for pulmonary infections. The performance of postoperative pulmonary infection varies greatly among different patients, and is affected by multiple factors such as neurogenic pulmonary oedema, swallowing dysfunction leading to aspiration, and prolonged bed rest, with complex associations among the factors. Previous studies have analysed individual factors in isolation and lacked comprehensive and systematic studies. Therefore, in-depth dissection of pulmonary infection risk factors and construction of accurate prediction models are of urgent and important significance for improving postoperative outcomes and enhancing clinical treatment of patients with basal ganglia haemorrhage.

### 4.1 Independent risk factor analysis

This study focuses on the in-depth analysis of risk factors for postoperative pulmonary infection in patients with basal ganglia haemorrhage and the construction of a prediction model, aiming to provide a precise basis for clinical control. In this study, a rigorous statistical analysis and multifactorial consideration confirmed that smoking history, duration of ventilator use, preoperative endotracheal intubation, preoperative vomiting, and preoperative GCS scores, were independent risk factors for postoperative lung infections in patients with basal ganglia haemorrhage.

Prolonged smoking impairs lung physiology and immune function in many ways, which significantly increases the risk of postoperative lung infections in patients with basal ganglia haemorrhage. In terms of physiological structure, nicotine, tar and other harmful substances in tobacco can cause airway epithelial cell chemotaxis, cilia shortening, inverted or even falling off, weakening the mechanical clearance ability of the respiratory tract to pathogens. At the same time, the destruction of alveolar walls and the decline in lung elasticity and retraction lead to ventilation dysfunction, making it easier for the lungs to retain pathogens. From the perspective of immune function, smoking inhibits the phagocytic activity of alveolar macrophages, reduces immunoglobulin secretion, and lowers the local immune defence capacity of the lungs. The incidence of infections varied significantly between patients with different smoking intensity and duration. The incidence of postoperative lung infections was much higher in patients who were heavy smokers (more than 20 cigarettes per day) and had smoked for a long time (more than 20 years) than in patients who were light or short-term smokers (23, 24). The uniqueness of smoking compared to other risk factors is that its long-term chronic damage to the lungs is persistent, with structural and functional changes in the lungs persisting even some time after cessation of smoking, increasing the risk of infection (25). In addition, prolonged use of ventilators increases exogenous infections mainly through several mechanisms. Bacterial biofilm formation is one of the key factors. The warm, humid environment inside the ventilator tubing is suitable for bacterial adhesion and growth, and biofilms gradually form. Bacteria in the biofilm are more resistant to antibiotics and continuously release planktonic bacteria, which become a source of persistent infection. At the same time, the positive pressure effect during ventilator ventilation tends to damage the airway mucosa, destroying the natural barrier of the respiratory tract and making it easier for pathogens to invade lung tissue (26, 27). Not only that, this study confirms that tracheal intubation can seriously damage the respiratory barrier function. During intubation, the tracheal tube directly damages the airway mucosa, blocking cilia movement and preventing effective removal of respiratory secretions and pathogens. At the same time, mucosal integrity is disrupted, providing a pathway for bacterial invasion. The longer the intubation time, the more severe the damage to the respiratory tract and the higher the risk of postoperative lung infection. Studies have shown a significant increase in the incidence of infection after more than 48 h of intubation. Intubation technique is also closely related to the risk of infection. Rough intubation is likely to cause mucosal tearing and bleeding, increasing the chance of infection (28, 29). Therefore, optimising the intubation process in the clinic can effectively reduce the risk of infection. Before intubation, the patient’s airway condition should be fully assessed, an appropriate diameter tracheal tube should be selected, and gentle and precise intubation techniques should be used to reduce mucosal injury. In the study, the pathophysiological process of preoperative vomiting triggering misaspiration, which in turn initiates lung infection, was also explored. During vomiting, stomach contents reflux into the respiratory tract, in which gastric acid and pepsin are strongly irritating to lung tissues, which can directly damage alveolar epithelial cells and airway mucosa, and destroy the defence barrier of the lungs. At the same time, a large number of bacteria carried by gastric contents enter the lungs and multiply rapidly in a suitable environment, triggering infections (30, 31).

Secondly, the GCS score is closely related to the severity of the patient’s condition and the immune status of the body. The GCS score mainly evaluates the patient’s state of consciousness from the three aspects of eye-opening response, speech response and limb movement, and the lower the score, the worse the patient’s condition is. Patients with severe disease often have dysregulation of the body’s stress response and suppression of immune system function. On the one hand, the increased secretion of glucocorticoids under stress inhibits the function of immune cells, such as the proliferation of lymphocytes and the decrease in phagocytic activity of macrophages; on the other hand, patients may have nutritional and metabolic disorders, which further weaken the immune function (32, 33). Therefore, when stratifying the risk of postoperative infection for patients, a comprehensive assessment can be made by combining the GCS score and other factors, such as age and underlying diseases. For patients with low GCS scores, they should be highly alert to the risk of pulmonary infection, and postoperative monitoring and care should be strengthened, such as close observation of respiratory conditions and regular lung imaging.

### 4.2 Exploring the clinical value of predictive modelling

Based on the in-depth analysis of the risk factors of postoperative lung infection in patients with basal ganglia haemorrhage, the prediction model constructed in this study has important clinical value. In terms of model accuracy assessment, after in-depth analysis of the training set and validation set, the model showed good sensitivity and specificity, and was able to identify potentially infected patients with greater accuracy. The ROC curve for the training set of this study, with an AUC of 0.888 (95% CI = 0.846–0.929), and the validation set, with an AUC of 0.853 (95% CI = 0.780–0.925), suggests that it can provide a reliable basis for risk judgement in different clinical scenarios, such as a large general hospital versus a primary healthcare facility. In terms of model application cases, in actual clinical practice, through the model, doctors successfully identified a patient with basal ganglia haemorrhage of advanced age and with multiple underlying diseases as a high-risk group for lung infection at an early stage. Based on the results of the model, the doctor adjusted the treatment plan in advance, shortened the time of ventilator use, and strengthened respiratory management. Eventually, the patient’s postoperative lung infection was effectively prevented and the recovery process was significantly accelerated. This fully demonstrates the advantages of the model in guiding clinical decision-making with early and precise warnings and assisting in the formulation of personalised treatment plans. However, the model also has certain limitations, such as the accuracy may be affected when facing complex and rare cases. Compared with the existing prediction tools at home and abroad, the accuracy of this model is significantly improved, and the AUC of this model is 0.888. This model is more convenient to operate, without complex calculations and a large number of data inputs, and it has a good universality in different geographical areas and hospitals of different levels. Its innovation lies in the incorporation of several key factors that were easily overlooked in previous studies, such as preoperative vomiting and detailed smoking history characteristics, which greatly improved the prediction effect. This advantage gives the model great potential for clinical application and is expected to be a powerful tool for clinicians to prevent and control postoperative lung infections in patients with basal ganglia haemorrhage.

### 4.3 Comparison with previous studies and in-depth discussion of the clinical significance of the study’s existence

The study team reviewed a large amount of literature, and in terms of risk factors, the independent risk factors identified in this study, such as smoking history, duration of ventilator use, preoperative endotracheal intubation, preoperative vomiting, and preoperative GCS scores, were partially consistent with previous studies. Ruediger Hilker et al. have clearly pointed out that smoking can cause damage to the structure and immune function of lungs, and increase the risk of infections. This study further refined the association between different smoking intensity and duration and the incidence of postoperative lung infections (13). Afshin et al. study pointed out on the duration of ventilator use and also mentioned that prolonged use of ventilator is prone to exogenous infections (7). This study, on the other hand, provides insight into the specific mechanisms of bacterial biofilm formation and airway mucosal damage. However, the factor of preoperative vomiting, which has been less frequently explored in depth as an independent risk factor in previous studies, was found to initiate the pathophysiological process of lung infection by triggering aspiration. This difference may stem from the different characteristics of the subjects in different studies, such as the differences between the group of patients included in this study and previous studies in terms of the severity of the disease and the distribution of the underlying disease, or the differences in the methodology and sample size of the studies, which lead to different sensitivities to this factor.

In addition, previous prediction models for postoperative lung infection after basal ganglia haemorrhage were mostly constructed using traditional statistical methods. However, the accuracy for precisely identifying high-risk patients is poor. The present study benefited from the inclusion of several key factors that were easily overlooked in the past in terms of improved prediction accuracy, and the expanded scope of application was reflected in the better prediction results for patients with different levels of illness and in different hospital settings. Lessons learned from previous studies in terms of variable selection and model validation were drawn to continuously optimise the model in this study. The clinical significance of this study is significant, as the clear independent risk factors provide a clear target for clinicians to assess the risk of patients, and the accurate prediction model helps to identify high-risk patients at an early stage, and take targeted preventive and interventional measures, such as rationally adjusting the strategy of ventilator use and optimising the preoperative preparation process, thus reducing the incidence of postoperative lung infections in patients with basal ganglia haemorrhage, improving the patient’s prognosis, and reducing the burden of healthcare.

### 4.4 Limitations of the study and exploration of future research directions

Although this study has achieved some results in the construction of risk factors and prediction models for postoperative pulmonary infection in patients with basal ganglia haemorrhage, there are inevitably some limitations. Regarding the sample, the sample size of this study is relatively limited, which may lead to a lack of generalisability and representativeness of the findings, making it difficult to fully cover the differences in the characteristics of patients in different geographical areas and medical conditions, as the literature points out the influence of the sample source and the sample size on the extrapolation of the results of the study. This limitation can be improved in the future by expanding the sample size through multi-centre studies, uniting hospitals in different regions and levels, and including a wide range of patients. In terms of research methodology, the data collection process may result in missing or inaccurate information due to incomplete recording of some medical records or subjective judgement bias; When variables are measured, some factors such as the quantification of patients’ smoking history and the determination of indicators of the body’s immune function may be in error; The duration of this study was relatively short, so some clinical data related to postoperative pulmonary infection following basal ganglia haemorrhage may not have been observed. Future studies should collect clinical data from patients over a longer period of time to improve the accuracy of the prediction model. The follow-up time of this study is relatively short months, which makes it difficult to comprehensively observe the long-term incidence of postoperative lung infections and the related long-term effects. Follow-up studies should extend the follow-up period to obtain longer-term clinical data to provide more time-sensitive clinical guidance. In this study, through the in-depth analysis of the risk factors of postoperative pulmonary infection in patients with basal ganglia haemorrhage, we identified several independent risk factors and constructed a prediction model with certain clinical value. The research results provide an important basis for clinicians to assess patient risk and identify high-risk patients at an early stage. In addition, this study only classified variables into two categories. Certain indicators may be more sensitive to the impact of basal ganglia haemorrhage on postoperative pulmonary infection. For example, different smoking statuses may have different effects on the results. Therefore, future studies should conduct more detailed subgroup analyses to fully investigate the impact of different variables on postoperative pulmonary infection following basal ganglia haemorrhage. This study excluded patients who died during the study period, which may have introduced a certain degree of bias, especially when patient deaths may have been related to pulmonary infection. Future research should combine multidisciplinary consultations to thoroughly analyse the causes of death in patients, with a view to minimising the impact of bias. Although this paper has made every effort to exclude the influence of confounding factors, due to the nature of retrospective studies, it is still inevitably affected by confounding factors. Future research should clearly define the influence of each variable and collect and evaluate in detail the risk factors that may affect postoperative pulmonary infection after basal ganglia haemorrhage surgery in order to minimise the influence of confounding factors.

## 5 Conclusion

In this study, a rigorous statistical analysis and multifactorial consideration confirmed that smoking history, duration of ventilator use, preoperative endotracheal intubation, preoperative vomiting, and preoperative GCS scores, were independent risk factors for postoperative lung infections in patients with basal ganglia haemorrhage. The predictive model constructed in this study provides a highly valuable tool for clinical practice. With this model, clinicians are able to accurately identify patients at high risk of postoperative lung infections at an early stage. This advantage is significant in that it allows physicians to develop and implement targeted prevention and intervention strategies based on the patient’s specific situation before lung infection occurs, effectively reducing the incidence of postoperative lung infections in patients with basal ganglia haemorrhage and improving the prognosis of the patient.

## Data Availability

The raw data supporting the conclusions of this article will be made available by the authors, without undue reservation.
